# The Use of Free Fibula Flap in Different Extremities and Our Clinical Results

**DOI:** 10.7759/cureus.47450

**Published:** 2023-10-22

**Authors:** Numan Atilgan

**Affiliations:** 1 Department of Hand Surgery, Sanliurfa Mehmet Akif Inan Training and Research Hospital, Sanliurfa, TUR

**Keywords:** flap, nonunion, extremity, osteocutaneous, fibula

## Abstract

Background and objectives

Plastic, orthopedic, otolaryngology, and oromaxillofacial surgery specialists rely on fibula grafts to solve reconstructive problems. The aim of this study is to discuss the use and results of vascular fibula flaps in the treatment of bone and soft tissue defects in various regions with different etiologies.

Materials and methods

In our clinic, we treated 32 patients with osteocutaneous fibular flaps due to bone and soft tissue defects of different etiologies and varying anatomical regions. In our study, age, gender, side, cause of injury, surgical technique, treatment results, and complications were evaluated for each patient.

Results

Of the 32 patients, 25 were male, and 7 were female. The average age is 37.2 (27-56). The mean bone defect size was 10.45 cm. Bone defect occurred in eight patients due to osteomyelitis, eleven patients due to gunshot wounds, nine patients due to pseudoarthrosis, and four patients due to a giant cell tumor. We applied osteocutaneous fibula flap in 27 patients and vascularized fibular flap in five patients. Bone union could not be achieved in four patients, and bone grafting was performed as a secondary surgery. Local infection occurred in five patients, and their treatment was completed with debridement and antibiotic administration. Wound complications occurred in three patients at the donor site, which were treated with debridement and skin grafting. The mean duration of radiological union was three months, and complete union was achieved in the seventh month.

Conclusions

We have shown in our case series that free vascularized fibula transfer has gained an important place in the field of skeletal reconstruction and is a reliable method for various bone reconstructions.

## Introduction

In addition to being technically challenging, vascular fibula grafting is an extremely useful salvage option for limb reconstructions. Fibula graft indications are broad, and surgical techniques are also variable. Plastic, orthopedic, otolaryngology, and oromaxillofacial surgery specialists rely on fibula grafts to solve reconstructive problems [1,2].

The free vascularized fibula graft is widely used to reconstruct bone defects larger than 6 cm, considering its smooth shape, bone strength, pedicle length, and remodeling capacity in long bones. Most frequently, it is used in tumor surgery, as a savior in pseudoarthrosis, in the reconstruction of mandible defects, in the treatment of chronic osteomyelitis, in avascular necrosis of the femoral head, as a replacement therapy in leg length inequalities and long bone defects [1,2]. It can be used not only as a pedicle bone graft but also as a musculo-osteocutaneous graft by including the soleus muscle for forearm defects [3]. Unvascularized grafts cannot be remodeled, and the transplanted bone cannot fuse with the recipient bone [4]. Only a very small number of viable osteocytes can survive below the periosteum, and most undergo necrosis [5]. If the bone defect is more than 5 cm, the use of avascular bone graft is contraindicated [6]. In vascular grafts, it is known that most cells remain alive, the bone can remodel, and the graft and the recipient bone can integrate, as in fracture healing [4].

Although the iliac bone, scapula, femur, rib, and radius have been used successfully for upper extremity reconstruction, the fibula has become the most appropriate choice for upper extremity reconstruction [7]. Vascular fibula transfer will be a more appropriate option for previous surgery in bone defects larger than 6 cm or for defective pseudoarthrosis with impaired blood supply secondary to infection [8]. With the vascular fibula graft, many surgeons have achieved significant union within an average of four to six months [9].

The fibula flap has a direct arterial supply from the peroneal artery. The artery length required for microsurgical anastomosis is ≤4-6 cm. The diaphyseal bone of the fibula has an endosteal and periosteal blood supply, and its endosteal vascularity comes from a single feeding artery, often considered the dominant pedicle [5].

The blood supply to the fibula is provided by three main vessels: anterior tibial, peroneal, and posterior tibial arteries. It should be determined before the procedure that the dorsalis pedis artery is the dominant vessel. Since foot ischemia will occur when a vascularized fibula graft is taken from the dominant artery, the peroneal artery, it should be known that it is a contraindication for the procedure [10].

The aim of this study is to discuss the use and results of the vascular fibula graft in the treatment of bone and soft tissue defects in various regions with different etiologies.

## Materials and methods

This study was conducted in accordance with the Declaration of Helsinki and approved by the Committee of Ethics of Harran University, Faculty of Medicine (HRÜ/22.16.28).

This retrospective study was performed using the data of 32 patients in the Training and Research Hospital between 2020 and 2022. Age, gender, side, cause of injury, surgical technique, results, and complications were evaluated for each patient.

Vascular perfusion assessment of the patient's lower extremity should be performed before surgery. For this purpose, duplex ultrasonography and computed angio-tomography are the most commonly used methods. With these methods, the posterior and anterior tibial arteries can be evaluated, as well as the perfusion of the peroneal artery. In peroneal artery insufficiency, fibula grafting should be abandoned, and alternative methods should be evaluated. Surgical procedures are performed by two teams. The first team works on the recipient site, while the other team works on the donor site. The procedure is done under general anesthesia. Pre-induction antibiotic prophylaxis is administered. The donor and recipient site are prepared with an antiseptic. Before the tourniquet is inflated, the blood in the lower extremity is emptied with the Esmarch. The tourniquet is inflated to 150 mmHg above the mean arterial pressure. Since the operation time of the patient may take six to eight hours, the contact areas of the shoulder, opposite fibula head, pelvis, and heels should be supported.

Debridement for the recipient site preparation, resection if a tumoral lesion is present, and frozen lymph node dissection can be performed. The proximal and distal periosteum should be preserved for bone union. After the procedures at the recipient site are completed, bone, soft tissue, and skin defects should be calculated, and the donor site team should be informed. Vessel diameter and pedicle length should also be calculated before flap removal [11].

**Figure 1:**
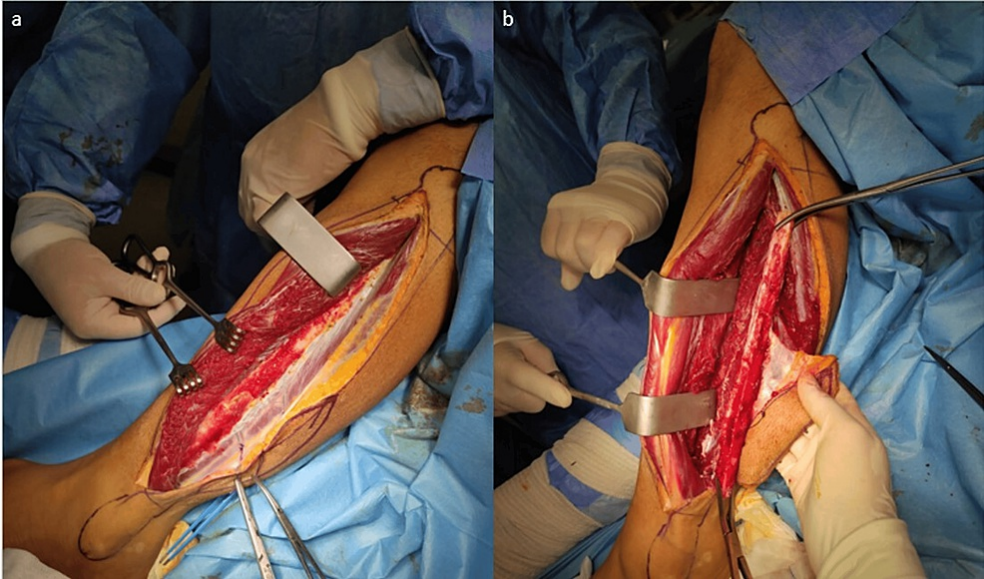
Fibula graft removal with an anterolateral approach. (a) Fibula graft removal is usually done with an anterolateral approach as described by Gilbert. (b) After careful retraction, the graft was removed.

Fibula graft removal is usually done with an anterolateral approach as described by Gilbert [12] (Figure 1 a,b).

The dissection is started between the peroneus longus and soleus muscles and advanced toward the fibula, the superficial peroneal nerve should be preserved. If a fibula graft is taken as long as possible, it can be prepared in the appropriate contour and length on the table before grafting. If there is an inequality in diameter between the fibula and the recipient bone, the fibula is divided so that the periosteum of the fibula is preserved and grafting can be achieved with the double-barrel technique. Fixation can usually be done with plates and screws or an external fixator. Vascular anastomosis is performed under the microscope with 8-0, 9-0 nylon sutures. While closing the donor site, the flexor hallucis longus is sutured to the interosseous membrane and the peroneus longus is sutured to the soleus. If possible, the skin is sutured with a primer, if it is not end-to-end, it can be closed with a skin graft [13]. If a skin graft has been applied to close the donor site, a dorsally supporting splint is applied and weight bearing is prevented for 48-72 hours. At the end of this period, walking boots are applied, plantar or dorsiflexion of the ankle is prevented, and if skin graft is applied, it will support the healing of the graft. At this moment, the patient is allowed to walk with as much weight as he can tolerate. To prevent deep vein thrombosis postoperatively, low-weight heparin and aspirin should be given for two weeks. If an osteocutaneous flap is used, the viability of the bone graft can also be evaluated by following the skin flap after the surgery [1].

Statistical analyses were performed with the IBM SPSS Statistics for Windows, Version 18 (Released 2009; IBM Corp., Armonk, New York) program. As descriptive statistics, arithmetic mean ± standard deviation and median (minimum, maximum) were used to summarize numerical data, and numbers and percentages were used to summarize categorical data. The relationship between categorical data was analyzed with the Chi-square (χ^2^) test. P-values below 0.05 were considered statistically significant.

## Results

Of the 32 patients, 25 were male and 7 were female. The average age is 37.2 (27-56). The mean bone defect size was 10.45 cm. Bone defects occurred in eight patients due to osteomyelitis, eleven patients due to gunshot wounds, nine patients due to pseudoarthrosis, and four patients due to giant cell tumors. We applied osteocutaneous fibula flap in 27 patients and vascularized fibular flap in five Patients (Table 1).

**Table 1:** Sociodemographic, trauma, and surgery flap type information of patients

	N (%) or median (min-max)
Gender	
Male	25 (78.1%)
Female	7 (21.9%)
Age	37.2 (27-56)
Trauma type	
Bone defect	8 (25.0%)
Gunshot wounds	11 (34.8%)
Pseudoarthrosis	9 (28.1%)
Giant cell tumor	4 (12.5%)
Flap type	
Osteocutaneous fibula flap	27 (84.4%)
Vascularized fibular flap	5 (15.6%)

Bone union could not be achieved in four patients, and bone grafting was performed as a secondary surgery. Local infection occurred in five patients, and their treatment was completed with debridement and antibiotic administration. Wound complications occurred in three patients at the donor site, which were treated with debridement and skin grafting. The mean duration of radiological union was three months, and complete union was achieved in the seventh month. Intraoperative thrombosis occurred in one patient; embolectomy and reanastomosis were performed. Finger part necrosis developed in one patient. A 3.5 mm dynamic compression plate (DCP) was used for bone fixation in five patients, a recon plate for two patients, and a DCP and external fixator for two patients.

**Figure 2:**
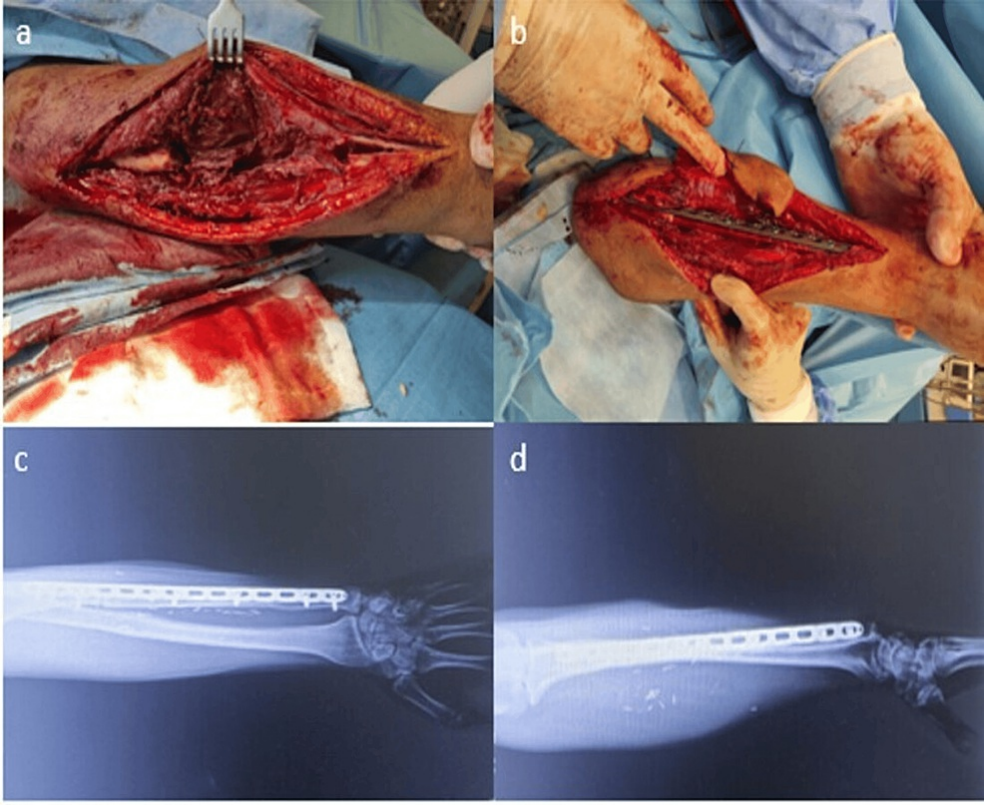
Osteocutaneous fibula flap application to a patient with a gunshot wound to the left ulna (a) A 12 cm bone defect in the ulna of the forearm. (b) An osteocutaneous vascular fibula was placed in the defective area of the ulna. (c) An AP film image of fusion in the defective area of the ulna. (d) The defective area of the ulna is fused with the vascular fibula.

In our fifth patient, a 38-year-old male, we applied an osteocutaneous fibula flap three weeks after debridement to address a 12 cm bone and soft tissue defect due to a gunshot wound to the left ulna. Ulnar artery end-to-end, deep vein, and superficial vein anastomosis were performed. An uncomplicated union was achieved in the third month (Figure 2 a-d).

**Figure 3:**
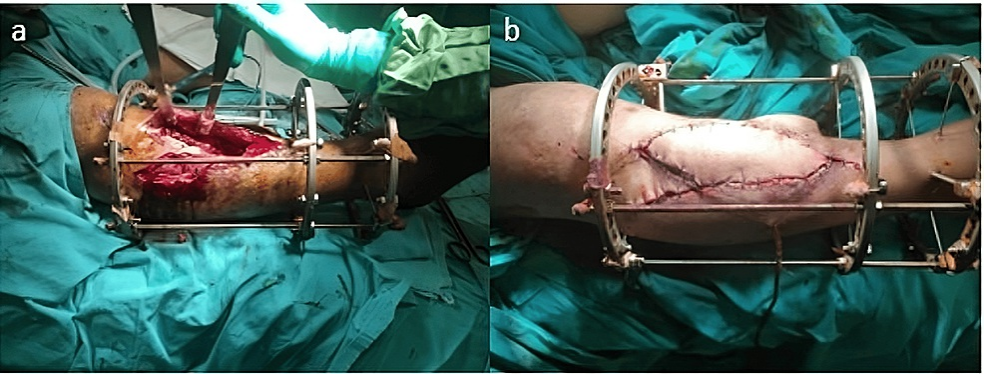
The application of an osteocutaneous fibula flap to a patient with a right tibial gunshot injury. (a) The prepared version without placing the osteocutaneous fibula in the defective area of the tibia. (b) The osteocutaneous fibula as it is placed on the tibia.

Our 41-year-old male patient developed a 12 cm bone and soft tissue defect in the proximal tibia after a gunshot wound to the right tibia, and an osteocutaneous fibula flap was applied four months after debridement. Debridement was performed after the infection occurred in the seven-month follow-up. Union was achieved in the fifth month after tibialis posterior artery anastomosis and deep vein anastomosis (Figure 3 a,b).

## Discussion

In our study, we have demonstrated in our case series that free vascularized fibula transfer has gained an important place in the field of skeletal reconstruction and is a reliable method for various bone reconstructions. We fixed the vascularized fibula graft with a plate and an external fixator in our 35-year-old male patient with a distal humeral fracture. This approach was chosen because he had pseudoarthrosis with a 12 cm bone defect in the humerus, despite undergoing seven operations in other centers using bone grafts. Radiological union was achieved in our patient in the fourth month. Although this method is more invasive and riskier than classical osteosynthesis and grafting, its use as a salvage procedure by experienced surgeons is becoming increasingly common. When conventional treatment methods fail in humerus fractures, vascular fibula transfer can serve as a reliable method for achieving bone union. One major drawback of using vascular fibula grafts is their smaller cross-sectional diameter, especially in large bone reconstructions such as the femur, tibia, and humerus; therefore, weight-bearing is often delayed by the patient. Graft hypertrophy takes time, which may be unacceptable in patients with sarcoma, who may have a shorter life expectancy. In such cases, a double-barrel transplant or combined vascular fibula and allograft may be required.

In eight of our patients with osteomyelitis distal to the radius, we applied vascularized fibula flaps to four and osteocutaneous fibula flaps to the other four. This is discussed in the forearm section. The treatment of chronic osteomyelitis is challenging due to changes in the bacterial flora and the growth of resistant bacteria. Systemically administered antibiotics cannot effectively reach biologically inactive bone surrounded by infected tissues. In the presence of necrotic bone or foreign matter, bacteria form biofilms, creating a barrier to antibiotics and host defense mechanisms. Therefore, radical debridement of infected foci is the cornerstone of osteomyelitis treatment [14]. Bone defects following radical debridement of infected tissues require gradual treatment and can rarely be addressed in a single session [15]. After the infection subsides and the fracture stabilizes, a second-stage reconstruction should be planned to address the bone defect. In cases of osteomyelitis, tumors, or even higher-energy trauma, appropriate blood vessels for microsurgical tissue transfer may not be readily available, necessitating the use of an arteriovenous (AV) fistula to enhance the success of microvascular anastomosis [1,4].

In our 26-year-old male patient with a giant cell tumor in the distal left radius, tumor excision and an osteocutaneous fibula flap were applied to address the 13 cm defect in the same session. We performed radial artery end-to-end and cephalic vein anastomosis. Union was achieved in the fourth month postoperatively, and no complications were observed during the nine-month follow-up.

Vascular fibula grafts have very high bone fusion rates and can improve regional circulation, especially when the environment is damaged by chemotherapy and irradiation [16]. Minami et al. reported an impressive series of free vascularized fibula transfers for reconstruction of long bones after bone excision using the fibula itself as the intercalary material [17]. This method relies on two features that uniquely adapt the vascularized fibula to skeletal reconstruction: linear geometry and ability to hypertrophy when placed in a weight-bearing environment [18]. This technique has shown encouraging results for the reconstruction of bone tumors [19]. It shows great promise, especially for orthopedic oncology. While other vascularized bone transfers such as the radial forearm flap, scapula flap, and iliac wing flap are available for skeletal reconstruction, no other flap provides a large enough amount of linear bone to be transferred with minimal morbidity. However, it is probably not possible to transfer a vascularized fibula at the time of resection in every patient, given that physicians who, together with a microsurgeon, are likely to perform bone tumor excision or treat pathological fractures are not available at all centers.

We applied osteocutaneous vascular fibula graft to 12 patients (ten radius and two ulna defects) in our patient group. The mean radius bone defect was 11.4 cm, and the ulna bone defect was 12 cm. One of our patients, a 27-year-old male, was diagnosed with osteomyelitis after a radius fracture. We performed debridement and antibiotic cement application in a one-month period, then fixation was achieved with a vascular fibula graft. Radial artery end-to-end anastomosis cephalic vein anastomosis was performed. A complete union was achieved in the seventh month. One of our patients, a 47-year-old male, caused a left 12 cm bone and soft tissue defect after a gunshot wound to the left radius, so we applied an osteocutaneous fibular flap after debridement. We performed radial artery end-to-end, basilic vein anastomosis. No union was observed in the early follow-ups, union was achieved in the sixth month after the iliac wing was supported with bone graft. One of our patients, a 43-year-old male, who developed osteomyelitis after a 10 cm bone and soft tissue defect after a gunshot wound in the distal right radius, three times after debridement, an osteocutaneous fibula flap was applied. Radial artery end-to-end, cephalic vein superficial vein anastomosis was performed. Debridement was performed due to short-term discharge after the operation. Union was achieved in the third month. One of our patients, a 56-year-old male, had an 11 cm bone and soft tissue defect due to osteomyelitis after a gunshot wound to the left radius distal, and an osteocutaneous fibular flap was performed twice during debridement follow-up. The radial artery was end-to-end, and the cephalic vein was anastomosed to the superficial vein. Union was achieved without complications in the second month. In our fifth patient, a 38-year-old male patient, we applied an osteocutaneous fibula flap three weeks after debridement to a 12 cm bone and soft tissue defect due to a gunshot wound to the left ulna. Ulnar artery end-to-end, deep vein, and superficial vein anastomosis was performed. An uncomplicated union was achieved in the third month (Figure 2a-d).

Several small series have shown excellent results using the Masquelet technique for patients with a forearm less than 5 cm short and a single bone defect [20,21]. Treating nonunions due to segmental bone loss in the radius and ulna is challenging. Although techniques such as bone transport with Ilizarov exist, this technique does not work in avascular bone after infection [22-24].

Free fibula transfer is considered the most suitable autograft for tibia reconstruction, given its long cylindrical flat shape, mechanical strength, predictable vascular pedicle, and potential for hypertrophy. The cross-sectional area of the fibula graft is significantly smaller than that of the femur, making it more prone to complications of stress fractures when subjected to weight-bearing. When the graft is used in two parts with the preservation of a vascular pedicle, it can overcome this problem and also provide structural support by increasing vascularity [25-28]. The double-barreled fibular flap is recommended for femur and proximal tibia reconstruction when the defect size is less than 13 cm [29].

We applied an osteocutaneous fibula flap to four patients due to first metatarsal bone defects. Our first patient, a 32-year-old male, developed a bone and soft tissue defect in the left first metatarsal after a gunshot injury. We performed osteocutaneous vascularized fibular flap surgery ten days after debridement as the initial intervention. Tibialis anterior end-to-end and saphenous vein superficial vein anastomosis were performed. Intraoperative thrombosis occurred, necessitating embolectomy and reanastomosis. Union was achieved in the tenth month. The second patient, a 36-year-old male, sustained a 7 cm bone and soft tissue defect after a gunshot wound to the right first metatarsal bone. We applied an osteocutaneous fibula graft 12 days after debridement. Tibialis anterior end-to-end and saphenous vein superficial vein anastomosis were performed. After the operation, partial necrosis developed in the first finger, requiring debridement. Union was achieved in the tenth month. The third patient, a 29-year-old male, had a 7 cm bony skin defect after a gunshot wound to the right first metatarsal, so we applied an osteocutaneous fibula graft on the fifth day after debridement and fixed it with an external fixator. Tibialis anterior end-to-end and saphenous vein superficial vein anastomosis were performed. Union was achieved in the seventh month postoperatively. The fourth patient, a 54-year-old male, developed osteomyelitis in the first metatarsal due to diabetes, and because he had a 7 cm bone and soft defect, we applied an osteocutaneous fibula flap in the first week after debridement. The patient experienced flap loss during the follow-up.

Lykoudis et al. were able to provide both bone and soft tissue reconstruction with the single-stage fibular osteocutaneous reconstruction technique. They showed that with this technique, bone union is faster, the possibility of infection is less, and post-procedure bone loss is minimal [30]. The advantages of using this technique; It offers sufficient bone length for metatarsal defects of all sizes, multiple osteotomies can be made while preserving the pedicle, the fibula thickens after surgery due to its high cortical density and is strong enough to support the body weight, it can tolerate angular loads due to its triangular structure similar to the metatarsal anatomy, the skin color in the osteocutaneous component is matched by the color of the dorsum of the foot. It has the advantage of being compatible [31,32]. We also used osteocutaneous fibula flap for metatarsal bone defect and our results were consistent with the literature.

Limitations of the study

The limitations of the study are that it is retrospective and the sample size is small. However, our study is valuable in terms of the fact that little work has been done on this subject in Turkey and it supports future studies.

## Conclusions

In conclusion, we have shown in our case series that free vascularized fibula transfer has gained an important place in the field of skeletal reconstruction and is a reliable method for different bone reconstructions. We used the fibula flap as a salvage procedure in our cases. Although the number of our patients was small, we achieved successful results for the forearm, foot, humerus and tibia. The objective results of more comprehensive studies on this subject and the use of clinical guidelines will contribute to determining which patients will need vascularized fibula transfer to save their limbs and which will have better results with amputation.
